# Direct Laser-Heated Pedestal (Czochralski) Growth of Rare-Earth Zirconate *A*_2_Zr_2_O_7_ Single Crystals from Mixed-Oxide Precursors

**DOI:** 10.1021/acsomega.6c09265

**Published:** 2026-10-15

**Authors:** Filip Hájek, Jindřich Dvořáček, Milan Klicpera

**Affiliations:** 37740 Charles University, Faculty of Mathematics and Physics, Department of Condensed Matter Physics, Ke Karlovu 5, 121 16 Prague 2, Czech Republic

## Abstract

High-quality single crystals of refractory oxides are essential for fundamental investigations and advanced functional characterization, yet their preparation is frequently limited by demanding precursor processing and the extremely high temperatures required for crystal growth. Here, we present a practical, reproducible, and experimentally accessible protocol for the preparation of rare-earth zirconate *A*_2_Zr_2_O_7_ single crystals based on direct processing of mixed-oxide precursors using a laser-heated pedestal (modified Czochralski) technique. In contrast to conventional approaches employing fully reacted polycrystalline feed rods, the proposed route starts directly from homogenized stoichiometric oxide mixtures, substantially simplifying precursor preparation, therefore reducing processing time, energy consumption, handling steps, and opportunities for contamination. The manuscript focuses on the technological aspects of the preparation procedure, including precursor fabrication, optimization of crystal-growth parameters, processing considerations, as well as practical limitations, scope, and applicability of the method. The protocol was successfully applied to 11 selected *A*_2_Zr_2_O_7_ compositions (*A* = Y, La, Nd, Eu, Gd, Dy, Ho, Er, Tm, Yb, and Lu), producing centimeter-scale single-crystalline ingots suitable for subsequent materials research. Representative structural and microstructural characterization confirms phase purity, crystallinity, and near-nominal cation stoichiometry, while detailed structural properties of the individual compounds are reported elsewhere. The presented methodology provides a robust and experimentally accessible route for the preparation of high-melting oxide single crystals and may be readily transferable to other refractory oxide systems requiring localized high-temperature melt growth.

## Graphical abstract

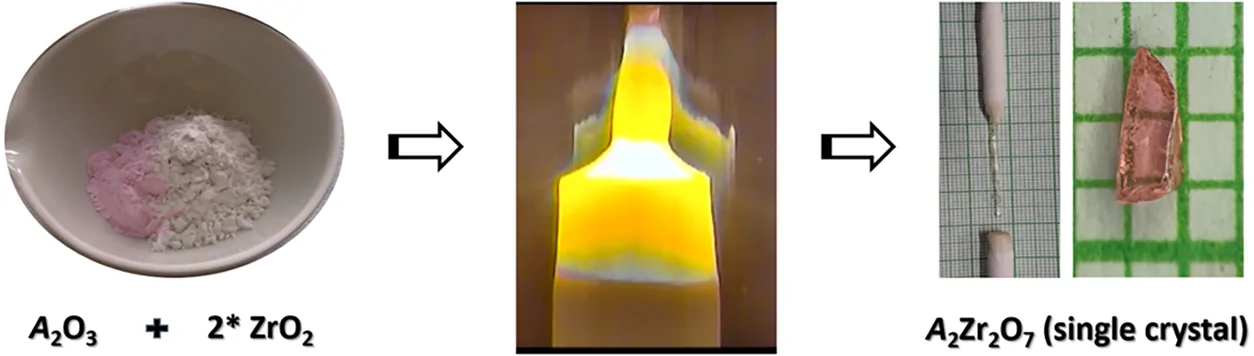

## 1 Introduction

Rare-earth zirconates with the general formula *A*_2_Zr_2_O_7_ constitute an important family of functional ceramic materials combining exceptional thermal and chemical stability with a broad range of technologically relevant properties. Depending on their composition, these compounds exhibit excellent radiation tolerance and long-term chemical durability required for nuclear waste immobilization,^1,2^ high oxygen-ion conductivity suitable for solid oxide fuel cells,^3,4^ electrical transport characteristics enabling thermistor applications,^5^ and low thermal conductivity attractive for thermal barrier coatings.^6,7^ Beyond these technological applications, rare-earth zirconates have also emerged as model systems for investigating geometrically frustrated magnetism, structural disorder, and correlated electronic phenomena.^8,9^

The crystallographic details of the *A*_2_Zr_2_O_7_ series are largely governed by the ionic radius ratio between the rare-earth and zirconium cations. Compounds containing larger rare-earth ions (La–Gd) crystallize in the ordered pyrochlore structure (space group *Fd*3̅*m*), whereas smaller rare-earth cations stabilize the defect-fluorite structure (space group *Fm*3̅*m*) because the reduced ionic-size mismatch promotes extensive *A*/Zr cation disorder.^10–13^ The Lu end member has recently been shown to adopt a lower-symmetry rhombohedral average structure,^11^ while local structural distortions extending beyond the average crystallographic symmetry have been identified throughout the heavy rare-earth members of the series.^14,15^ Owing to this structural diversity, rare-earth zirconates continue to attract considerable interest as model systems for studying disorder-driven phenomena and structure–property relationships.

Reliable investigation of these materials requires high-quality single crystals with well-controlled composition and minimal structural defects. Conventionally, polycrystalline *A*_2_Zr_2_O_7_ ceramics are synthesized by solid-state reactions involving repeated calcination, intermediate grinding, and prolonged high-temperature annealing.^16^ Alternative wet-chemical routes, including coprecipitation and sol–gel methods, have also been reported to improve chemical homogeneity or reduce reaction temperatures.^17^ Single crystals are most frequently grown by optical floating-hot-zone techniques from previously synthesized polycrystalline feed rods.^18–22,28^ Although these methods are well established, preparation of dense and chemically homogeneous polycrystalline precursors remains labor-intensive, time-consuming, and often requires multiple thermal cycles before material suitable for crystal growth is obtained.

The synthesis of zirconates presents an additional challenge because both starting oxides, zirconia and rare-earth sesquioxides, have exceptionally high melting temperatures and limited absorption of near-infrared radiation.^20^ These characteristics place stringent requirements on crystal-growth equipment and frequently restrict the applicability of conventional floating-zone techniques, particularly for heavy rare-earth members with the highest melting temperatures. Consequently, improvements in precursor preparation and crystal-growth methodology remain highly desirable from both practical and scientific perspectives, especially approaches capable of simplifying the overall preparation route while maintaining excellent crystal quality.

In our previous work, we demonstrated that high-quality *A*_2_Zr_2_O_7_ single crystals can be prepared directly from mixed-oxide precursors using laser-heated crystal-growth techniques, thereby eliminating the conventional synthesis of fully reacted polycrystalline feed rods.^23–25^ These studies established the feasibility of the approach and verified the structural quality of the resulting single crystals. The present work focuses on the technological aspects of this preparation route and provides a detailed experimental methodology for reproducible synthesis of rare-earth zirconate ceramics and single crystals. Particular attention is devoted to precursor fabrication, optimization of laser-heated Czochralski growth, practical processing parameters, and discussion of the advantages and limitations of the method relative to conventional preparation routes. Not only is the proposed methodology expected to be applicable to rare-earth zirconates, but also to a broad range of refractory oxide materials requiring crystal growth at extreme temperatures.

## 2 Synthesis of Ceramics and Single Crystals

### 2.1 Starting Materials

Rare-earth zirconates *A*_2_Zr_2_O_7_ (*A* = Y, La, Nd, Eu, Gd, Dy, Ho, Er, Tm, Yb, and Lu) were prepared from commercially available rare-earth sesquioxides *A*_2_O_3_ and zirconium dioxide ZrO_2_ (99.99% metal basis, Alfa Aesar). Prior to weighing, the oxide powders were dried at 200 °C to remove adsorbed moisture. Stoichiometric mixtures corresponding to the nominal composition *A*_2_Zr_2_O_7_ were prepared using an analytical balance with a resolution of 0.02 mg. Approximately 15 g of the powder mixture was weighed for preparation of each precursor rod.

The constituent oxides were subsequently homogenized by manual grinding and stirring in an agate mortar using an agate pestle. Particular attention was devoted to this step, as the present preparation route relies entirely on the initial homogeneity of the mixed oxides. In contrast to conventional solid-state synthesis, no repeated calcination and intermediate regrinding were performed prior to crystal growth. Consequently, thorough mixing of the starting powders represents the most critical step of the entire preparation procedure. Either dry or wet homogenization (using water, ethanol, or isopropyl alcohol followed by complete solvent evaporation) may be employed without affecting the subsequent crystal growth process.

### 2.2 Preparation of Precursor Rods

The homogenized powder mixture was transferred into cylindrical latex molds of approximately 10 mm diameter using a vacuum-assisted filling system. The powder was added gradually in small portions while being continuously compacted to maximize the packing density and minimize the amount of trapped air. At least two precursor rods were prepared simultaneously for each growth experiment. One rod was intentionally made longer to serve as the feed rod, whereas the second rod was shorter and subsequently machined into the seed rod.

The filled molds were sealed and manually shaped into cylindrical rods of nearly constant diameter below 10 mm (see Figure 1). The green bodies were then compacted under quasi-isostatic conditions using a custom-built pressure cell filled with mineral oil as the pressure-transmitting medium. A compressive load equivalent to approximately 1500 kg was applied, resulting in significant densification of the powder compact and removal of residual air from the interparticle volume. After pressing, the rods (approximately 8 mm in diameter) maintained their cylindrical shape and could be removed from the molds without damage, although their mechanical strength remained insufficient for subsequent machining and crystal growth.

**Figure 1:**
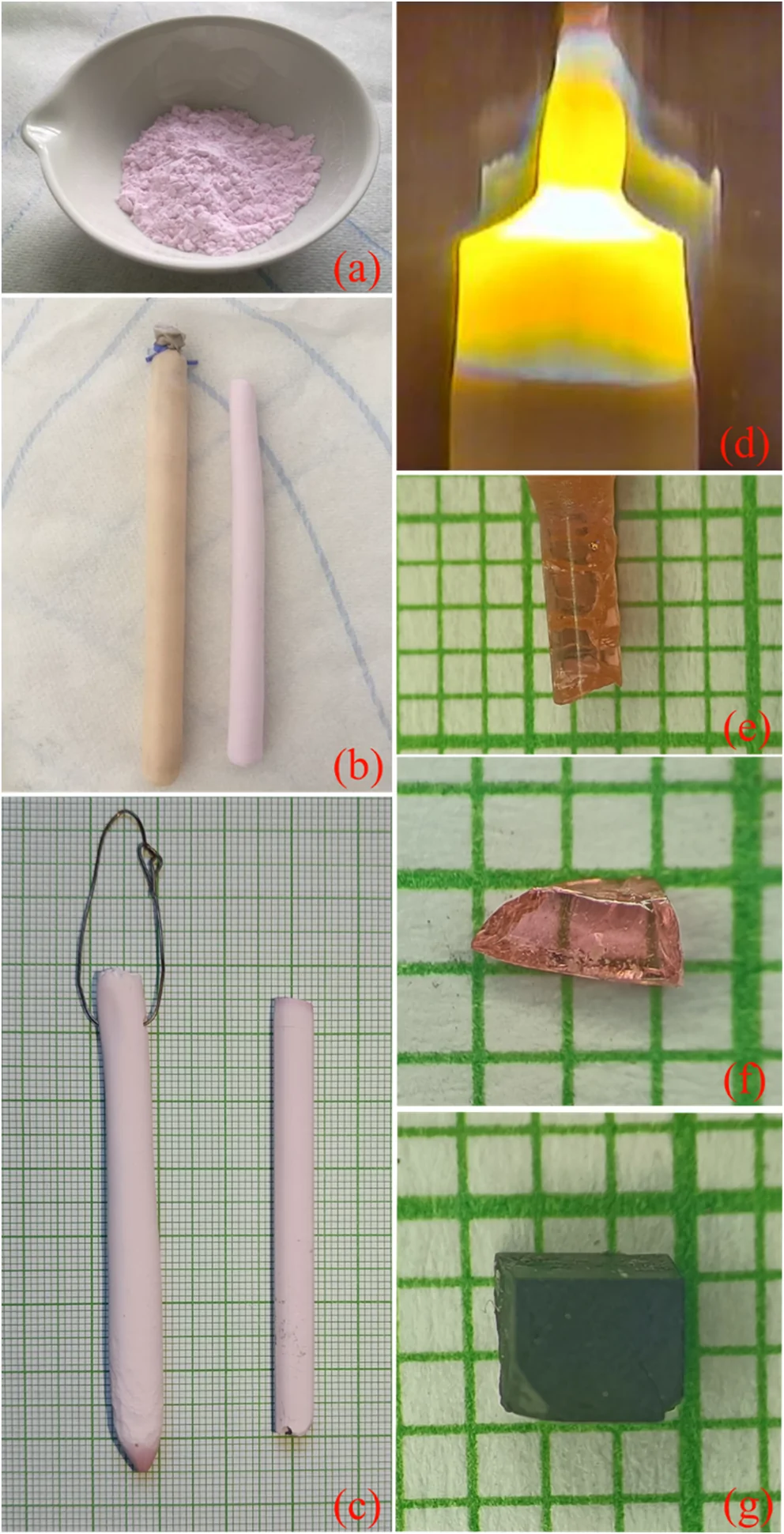
Representative stages of the preparation procedure for rare-earth zirconate single crystals grown directly from mixed-oxide precursors. (a) Homogenized stoichiometric mixture of erbium and zirconium oxides after grinding. (b) Green precursor rod after quasi-isostatic pressing in rubber form and extracted from the form. (c) Mechanically stabilized feed rod and conically machined seed rod after low-temperature presintering; prepared for growth in laser furnace. (d) Stable molten zone during laser-heated Czochralski crystal growth. (e) Representative as-grown single-crystalline ingot; cracks are visible in translucent crystals. (f) Cracks-free single-crystalline grain of Er_2_Zr_2_O_7_. (g) Example of oriented crystal piece, grown in Ar atmosphere, prepared for subsequent structural and physical-property measurements.

To improve their mechanical stability, the pressed rods were presintered in air at 850 °C for 4 h, with the standard heating/cooling rate of 5 °C/min. During this treatment, the contact area between neighboring particles increased through solid-state sintering, accompanied by only limited solid-state interdiffusion and partial reaction between the constituent oxides. The resulting precursor rods exhibited an approximately 10–15% volume reduction and sufficient mechanical integrity for further handling while remaining chemically close to the initial oxide mixture.

After sintering, the precursor rods were mechanically machined to ensure a uniform cylindrical geometry. The feed rod was polished at one end to obtain a flat upper surface, thereby faciliating efficient absorption of laser radiation and formation of a stable molten zone. The shorter rod was shaped into a conical seed, and a small hole was drilled at its opposite end to enable suspension from the upper shaft of the growth furnace. These modifications were found to improve the reproducibility of the crystal growth process and the stability of the molten zone during the initial stage of growth.

### 2.3 Laser-Heated Czochralski Crystal Growth

Single crystals were grown using a laser-heated pedestal system (CSC FZ-LD-5–200W–II–VPO-PC) equipped with five semiconductor laser diodes providing a maximum combined optical power of 1000 W. The actual power required depends systematically on the rare-earth member, with the heavier members requiring operation closer to the maximum available power (Table 1). The laser beams were focused onto the sample through individual optical lenses and inclined by approximately 30° with respect to the horizontal plane, creating a highly localized (approximately 4 × 4 mm^2^) molten zone with temperatures estimated to approach 2800 °C.

**Table 1:** Growth, Structural, and Compositional Parameters of the Investigated *A*_2_Zr_2_O_7_ Single Crystals^a^,^b^

*A*_2_Zr_2_O_7_	laser power (W)	*A*/Zr^EDX^ (%/%)	*r*_*A*3+_/*r*_Zr4+_	space group	*a* (Å)	*x*_48f_
Y	840	49(2):51(2)	1.42	*Fm*3̅*m*	5.208(1)	-
La	750	49(2):51(2)	1.61	*Fd*3̅*m*	10.819(2)	0.343(2)
Nd	790	50(2):50(2)	1.54	*Fd*3̅*m*	10.688(2)	0.346(2)
Eu	850	50(2):50(2)	1.48	*Fd*3̅*m*	10.579(2)	0.340(2)
Gd	900	49(2):51(2)	1.46	*Fm*3̅*m*	5.267(1)	-
Dy	920	50(2):50(2)	1.43	*Fm*3̅*m*	5.223(2)	-
Ho	930	50(2):50(2)	1.41	*Fm*3̅*m*	5.216(1)	-
Er	950	49(2):51(2)	1.39	*Fm*3̅*m*	5.201(1)	-
Tm	930	49(2):51(2)	1.38	*Fm*3̅*m*	5.185(1)	-
Yb	940	50(2):50(2)	1.37	*Fm*3̅*m*	5.160(1)	-
Lu	990	50(2):50(2)	1.36	*R*3̅	^b^	^b^

a Listed are the combined heating power of five laser diodes, rare-earth and zirconium contents determined by energy-dispersive X-ray spectroscopy (EDX), ionic-radius ratio *r*_A3+_/*r*_Zr4+_, crystallographic space group, refined lattice parameter *a*, and the free fractional coordinate of oxygen at the 48f Wyckoff position, *x*_48f_, for compounds crystallizing in the pyrochlore structure.

b Lu_2_Zr_2_O_7_ crystallizes in rhombohedral structure with hexagonal lattice parameters *a* = 9.661(2) Å and *c* = 8.961(2) Å, and fractional coordinates Lu/Zr in 3a positions (0, 0, 0), Lu/Zr in 18f^1^ position (0.256(1), 0.212(1), 0.347(1)), O^1^ in 6c position (0, 0, 0.463(9)), O^2^ in 18f^2^ position (0.046(1), 0.232(1), 0.388(1)), and O^3^ in 18f^3^ position (0.234(2), 0.168(2), 0.105(1)).

Crystal growth was carried out in air under elevated pressure (approximately 200 kPa gauge pressure) with continuous gas flow of 1.5 L/min. Selected compositions were also grown under flowing oxygen, yielding comparable crystal quality and growth stability. The growth under Ar atmosphere was attempted as well, leading to oxygen-deficient crystals (significantly darker crystals compared to those grown in air; compare Figure 1f,1g). For several of the investigated compositions, particularly those containing the heaviest rare-earth elements, the laser-heating system operated close to its maximum available power owing to the extremely high melting temperatures of both the constituent oxides and the resulting zirconates. In addition, the relatively low thermal conductivity and limited absorption of near-infrared radiation reduce the stability of the molten zone during crystal growth for the heavier rare-earth members of the series. Therefore, a laser-heated pedestal (modified Czochralski) technique was employed throughout this work.

During growth, the conical seed rod was gradually lowered until its tip made contact with the molten surface of the feed rod. After establishing a stable solid–liquid interface, the seed rod was slowly withdrawn vertically while both shafts rotated in opposite directions to improve mixing within the melt. The seed-pulling rate of 6.0 mm/h, feed-rod translation rate of 0.5 mm/h and counter-rotation speed of 10 rpm (5 rpm upper shaft, 5 rpm lower shaft) were found to provide stable growth conditions for most compositions investigated. Before reaching steady-state growth, minor adjustments of the pulling conditions were typically required to stabilize the diameter of the growing crystal. Once a stable molten zone was established, single-crystalline ingots with diameters of 2–3 mm and lengths of several centimeters were routinely grown.

Once sufficiently large ingot was synthesized or the precursor was fully used, the grown ingot was cooled down at a rate of 30 W/h down to room temperature. No postgrowth annealing treatment was applied to the as-grown crystals.

Unlike conventional crystal-growth procedures employing fully reacted polycrystalline feed rods, the present approach relies on direct melting of mixed-oxide precursors. The high diffusivity in the molten zone promotes rapid homogenization of the constituent oxides, allowing crystal growth directly from the initial mixture without prior synthesis of a single-phase ceramic precursor. Consequently, repeated calcination, intermediate grinding, and prolonged high-temperature annealing become superfluous.

### 2.4 Structural Characterization

The as-grown ingots were first examined by optical microscopy to identify regions suitable for structural characterization. Whole ingots, followed by selected fragments extracted from their central regions, were investigated by back-reflection Laue X-ray diffraction to verify their single-crystalline nature, crystallographic quality, and orientation. The crystal-to-detector distance was 39 mm and the exposure time was 90 s. Program CLIP^27^ was employed for indexing procedure.

For phase analysis, selected parts of each ingot were ground into a fine powder and characterized by laboratory powder X-ray diffraction using a Rigaku Miniflex diffractometer with Cu Kα radiation over the angular range 10–120° with a step size of 0.02°. The diffraction data were analyzed by Rietveld refinement employing the appropriate crystal structure models for the individual members of the *A*_2_Zr_2_O_7_ series. The obtained diffraction patterns (Figure 2) confirmed the formation of single-phase materials without detectable impurity phases. The refined diffraction data were further used to determine the lattice parameters and confirm the expected structural evolution from the pyrochlore through defect-fluorite to rhombohedral symmetry across the rare-earth series.

**Figure 2:**
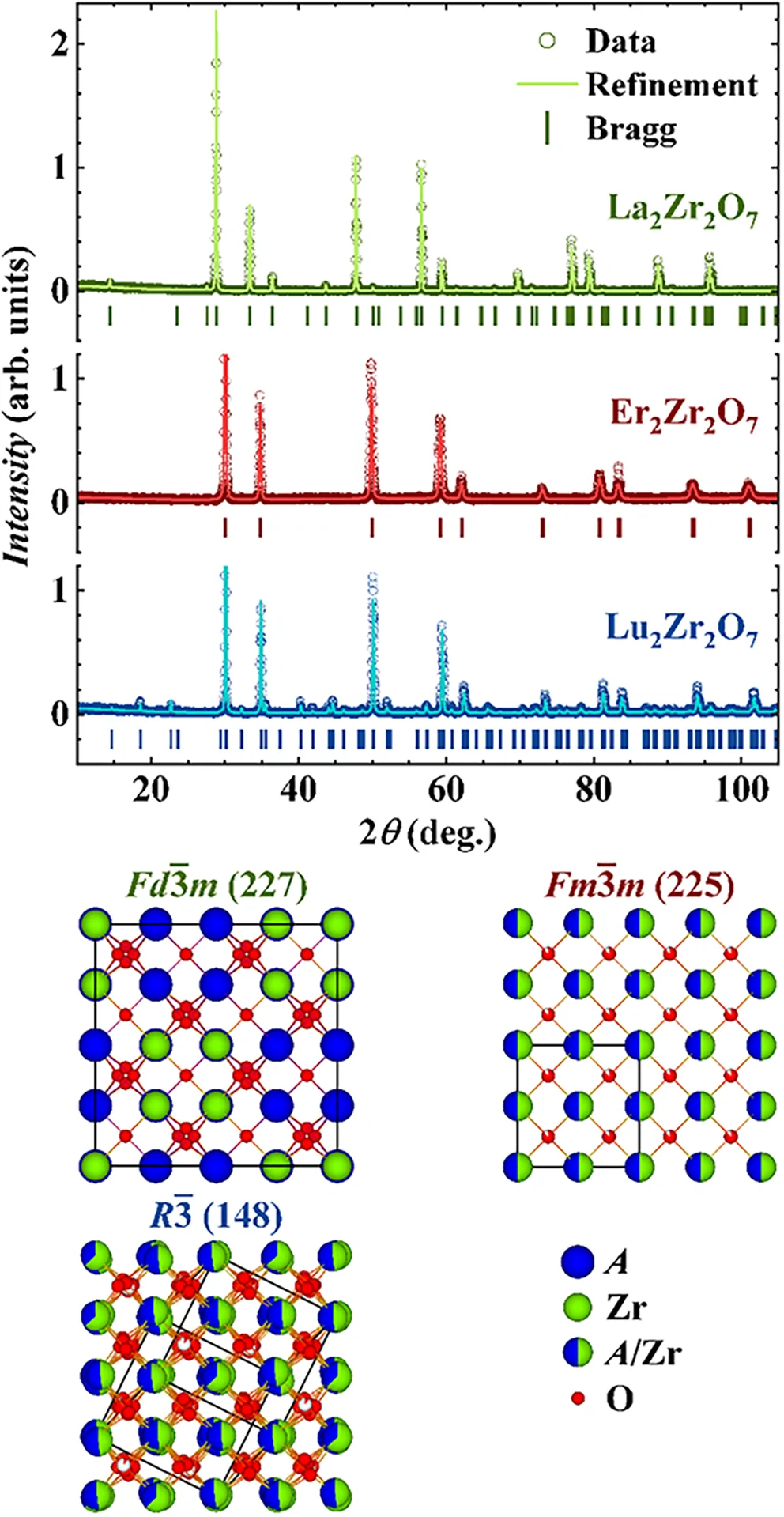
Powder X-ray diffraction patterns of selected *A*_2_Zr_2_O_7_ compounds covering the three average crystal-structure types observed across the rare-earth series. Symbols denote experimental data, solid lines represent the Rietveld fits, and vertical tick marks indicate Bragg reflection positions. For clarity, the most intense reflection at approximately 30° is truncated in the *A* = Er and Lu patterns to enhance the overall visibility of weaker diffraction peaks. The diffraction patterns confirm phase purity and the expected structural evolution from the pyrochlore (*Fd*3̅*m*) through the defect-fluorite (*Fm*3̅*m*) to the rhombohedral (*R*3̅) structure. Individual crystal structures are illustrated in corresponding figures. The crystallographic projections are selected to better reveal the differences/similarities among the three structures; the unit cells are highlighted.

## 3 Results and Discussion

### 3.1 Formation of Precursor Rods and Crystal Growth

The preparation procedure described above proved to be highly reproducible for the investigated rare-earth zirconate compositions. Homogeneous precursor rods with sufficient mechanical strength were routinely obtained after the low-temperature presintering treatment. Although the thermal treatment was intentionally limited to preserve the mixed-oxide character of the precursor, the rods exhibited adequate integrity for subsequent machining and handling during crystal growth. No cracking nor disintegration of the rods were observed during mounting into the growth furnace.

Following the laser heating, a stable molten zone could be established for all investigated compositions. The formation of the melt was accompanied by rapid homogenization of the constituent oxides, eliminating the need for prior preparation of a fully reacted polycrystalline feed rod. After optimization of the laser power and pulling conditions, the molten zone remained stable throughout the entire crystal-growth process, allowing continuous growth of dense single-crystalline ingots. Representative examples of the precursor rods, molten zone, and as-grown crystals are shown in Figure 1.

The growth behavior was found to depend systematically on the rare-earth element. Members containing larger rare-earth ions generally exhibited a broader processing window and greater molten-zone stability, whereas compounds containing the heaviest rare-earth elements required operation closer to the maximum available laser power owing to their higher melting temperatures and lower absorption of incident infrared radiation. Nevertheless, once stable growth conditions were established, no qualitative differences in the morphology or macroscopic quality of the obtained crystals were observed across the investigated series.

The resulting ingots typically reached lengths of several centimeters with diameters between 2 and 3 mm. Their external surfaces were smooth and generally free from visible macroscopic cracks, indicating stable solidification during growth. Occasional cracks were observed in individual ingots; however, crack-free sections suitable for subsequent structural and physical-property studies could be obtained even from such specimens (Figure 1e,f). Only minor variations of the crystal diameter were occasionally observed during the initial stage of growth while the thermal equilibrium of the molten zone was being established. After this transient period, the crystal diameter remained essentially constant over the entire growth length, reflecting the stable growth conditions and uniform geometry of the precursor rods.

A distinctive feature of the present approach is that crystal growth starts directly from mechanically stabilized mixed-oxide precursors rather than conventionally synthesized polycrystalline feed rods. The successful growth of all investigated compositions demonstrates that effective homogenization can be achieved within the molten zone prior to crystallization.

The reproducibility of the growth process was confirmed by repeated synthesis of several representative compositions, four independent growths of Er_2_Zr_2_O_7_ crystal and three growths of *A* = Eu, Tm, and Lu zirconates, yielding ingots with comparable dimensions and morphology under identical processing conditions. These observations demonstrate that the laser-heated Czochralski method employing mixed-oxide precursors represents a robust preparation route for rare-earth zirconates over a broad range of compositions.

### 3.2 Phase Purity and Structural Characterization

The phase purity and crystal structure of the synthesized materials were assessed by laboratory powder X-ray diffraction. Representative diffraction patterns illustrating the principal structural regimes encountered across the investigated *A*_2_Zr_2_O_7_ compositions are presented in Figure 2. All measured patterns could be satisfactorily described by the crystal structure models expected from previous studies,^12,23,26^ confirming successful synthesis of the target compounds without detectable secondary phases.

The light rare-earth members, La_2_Zr_2_O_7_, Nd_2_Zr_2_O_7_, and Eu_2_Zr_2_O_7_ were refined using the ordered pyrochlore structure (space group *Fd*3̅*m*), whereas the intermediate and heavy rare-earth compounds Y_2_Zr_2_O_7_ and Gd_2_Zr_2_O_7_–Yb_2_Zr_2_O_7_ were well described by the disordered defect-fluorite model (space group *Fm*3̅*m*). The Lu_2_Zr_2_O_7_ end member exhibited the rhombohedral structure (space group *R*3̅), in agreement with previous reports.^11^ The refined lattice parameters are listed in Table 1 and follow the expected systematic evolution across the investigated compositions.

No reflections attributable to secondary crystalline phases were detected within the instrumental resolution for any investigated composition. Minor discrepancies between calculated and observed intensities were occasionally present for several members. Similar deviations have previously been associated with local structural correlations extending beyond the average pyrochlore and defect-fluorite model^15^ rather than with impurity phases. Since these local distortions have already been investigated in detail using synchrotron and neutron total scattering techniques,^14^ they are not discussed further here.

The refined lattice parameters follow the expected systematic decrease across the rare-earth series, consistent with the lanthanide contraction. Their values agree within experimental uncertainty with those reported previously for samples prepared by conventional ceramic routes, as well as by the floating-hot-zone preparation procedure,^20,23,26^ demonstrating that direct crystal growth from mixed-oxide precursors does not alter the average crystal structure of the resulting materials.

Back-reflection Laue X-ray diffraction further confirmed the single-crystalline character of the grown ingots. Typical Laue patterns obtained from representative crystals are shown in Figure 3. The diffraction spots are sharp and well-defined over the entire illuminated volume, indicating low mosaicity and the absence of significant crystal fragmentation.

**Figure 3:**
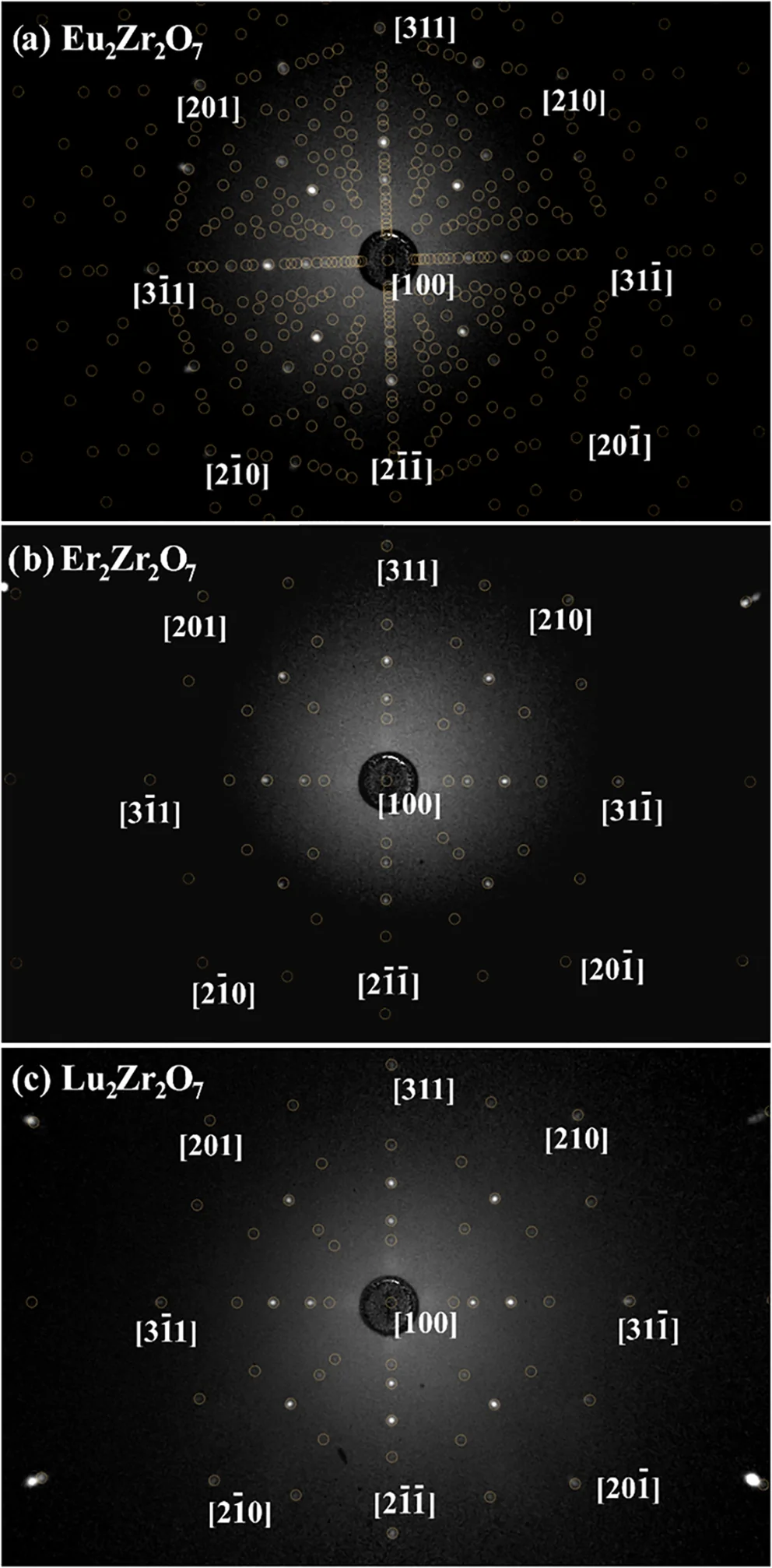
Back-reflection Laue diffraction patterns recorded from selected *A*_2_Zr_2_O_7_ single crystals (corresponding to Figure 2). The sharp diffraction spots confirm the high crystallographic quality of the grown single crystals. Calculated diffraction patterns are denoted by orange circles. (See separate high-quality pictures in uploaded files.) The principal directions [100] of (a) Eu_2_Zr_2_O_7_ pyrochlore, (b) Er_2_Zr_2_O_7_ defect-fluorite, and (c) Lu_2_Zr_2_O_7_ rhombohedral variant are indicated together with representative Miller indices.

Additional information on crystal quality was obtained by scanning electron microscopy combined with energy-dispersive X-ray spectroscopy (EDX). Backscattered electron images revealed homogeneous microstructures without detectable secondary phases or compositional segregation. Within the experimental uncertainty of the EDX method, the measured rare-earth-to-zirconium ratios agreed with the nominal stoichiometric composition for all investigated samples (Table 1). Although the oxygen content cannot be determined reliably by EDX, and quantitative analysis is further complicated by surface charging arising from the insulating nature of the crystals, no indication of cation off-stoichiometry was found.

Taken together, the diffraction and microscopy results demonstrate that the simplified preparation route consistently produces chemically homogeneous, phase-pure, and structurally well-defined rare-earth zirconate single crystals throughout the investigated compositional range. The structural characteristics are fully comparable with those reported for crystals prepared using conventional multistep precursor synthesis, confirming that omission of the polycrystalline precursor preparation does not compromise the quality of the final material.

### 3.3 Advantages of Crystal Growth from Mixed-Oxide Precursors

The principal advantage of the present preparation route is the elimination of conventional polycrystalline precursor synthesis prior to single-crystal growth. In standard preparation procedures, the desired *A*_2_Zr_2_O_7_ phase is first produced by repeated solid-state reactions involving multiple grinding, calcination, and homogenization steps before the material can be shaped into a feed rod. Although this approach reliably yields high-quality precursors, it requires substantial time, energy, and experimental effort. In contrast, the presented method employs mechanically stabilized mixed-oxide rods that are transformed directly into the final crystal during growth. Homogenization occurs in the molten zone rather than in the solid state, where diffusion processes are considerably slower. As a result, repeated calcination and intermediate grinding become excessive. See Table 2 for a direct procedural comparison between the conventional preparation of a fully reacted polycrystalline feed rod and the present mixed-oxide route.

**Table 2:** Comparison of the Conventional Polycrystalline Precursor Route and the Present Mixed-Oxide Precursor Route for *A*_2_Zr_2_O_7_ Crystal Growth^a^,^b^

Processing step	Conventional ceramic precursor route	Present mixed-oxide precursor route	Time (hours)
Starting materials	*A*_2_O_3_ + ZrO_2_	*A*_2_O_3_ + ZrO_2_	<0.5
Initial homogenization	Grinding and mixing of starting powders	Grinding and mixing of starting powders	>0.5
Solid-state reaction at high temperature	Repeated calcination with intermediate grinding to obtain single-phase *A*_2_Zr_2_O_7_ precursor	Not required	*n* × 4^b^
Precursor-rod forming	Filling of rubber mold	Filling of rubber mold	>0.5
Compacting rod	Quasi-isostatic pressing	Quasi-isostatic pressing	<0.5
Precursor-rod removal from rubber mold	Fragile rod requiring careful handling; spherical powder grains after previous heat-treatment and grinding	Mechanically robust rod; initial nonregular shape of grains of initial materials	<0.5
Precursor-rod heat treatment	Sintering to obtain a fully reacted ceramic precursor	Sintering for mechanical stabilization	4
Final precursor-rod state	Fully reacted, single-phase *A*_2_Zr_2_O_7_ ceramic; frequently fragile	Mechanically stabilized mixed-oxide precursor; usually robust	
Mechanical machining	Shaping the precursor	Shaping the precursor	<0.5
Total preparation time before growth	**23** ^b^	**7**	
Material losses before growth	Repeated handling and processing	Limited to single handling and machining	
High-temperature precursor treatments before growth	*n* + 1	1	

a The listed times represent approximate processing times before crystal growth; the conventional-route estimate is based on a minimum of four solid-state reaction cycles.

b For estimation of the minimum conventional preparation time, *n* = 4 is assumed; each reaction cycle is taken as 4 h. The actual number of cycles depends on the effect of solid-state synthesis procedure and the homogeneity achieved after each treatment.

The simplified workflow offers several practical benefits. Preparation time is significantly shortened, the overall thermal budget is reduced, and the number of processing steps is minimized. In addition, reduced handling lowers the probability of contamination originating from grinding media, reaction vessels or repeated transfer of powder between processing stages.

Another advantage is the more efficient utilization of starting materials. Conventional ceramic synthesis inevitably involves small material losses during repeated grinding, transfer, and shaping operations. In contrast, the present method incorporates the weighed oxide mixture directly into the precursor rods, thereby reducing material losses prior to crystal growth. This feature may be particularly beneficial when working with expensive rare-earth compounds or isotopically enriched materials.

The present results indicate that substantial simplification of precursor processing can be achieved without compromising the structural quality of the final material.

### 3.4 Applicability and Limitations of the Preparation Strategy

Although the present study focuses on the *A*_2_Zr_2_O_7_ rare-earth zirconate series, the proposed preparation strategy may extend beyond this particular family of compounds. The applicability demonstrated experimentally in the present work is restricted to the investigated *A*_2_Zr_2_O_7_ compositions; for other material systems, transferability remains to be established experimentally. The essential prerequisite is that both the starting materials and the target phase remain chemically stable under the high-temperature conditions required for melt growth. Materials satisfying these conditions may therefore be suitable candidates for direct crystal growth from mixed-oxide precursors, potentially replacing the conventional multistep preparation of polycrystalline feed rods.

The approach is expected to be particularly advantageous for refractory systems whose conventional preparation requires extensive solid-state processing. In such cases, transferring homogenization from the solid state to the molten phase can substantially simplify the overall experimental workflow while preserving control of the nominal composition.

The method may also prove useful in exploratory materials research. Since preparation of fully reacted ceramic feed rods is not required, new compositions can be tested rapidly by adjusting the composition of the initial oxide mixture. This capability may accelerate investigations of substituted compounds, compositional series, and newly designed materials where optimization of precursor synthesis would otherwise represent a significant experimental bottleneck.

The applicability of the method is nevertheless subject to the same fundamental constraints as other melt-growth techniques. The starting materials and the target compound must remain sufficiently stable at the temperatures required for crystal growth. Materials exhibiting substantial evaporation, incongruent melting, chemical decomposition or strong interactions with the growth atmosphere may require modified growth strategies or specialized furnace configurations.

Furthermore, successful crystal growth still depends on appropriate optimization of thermal gradients, pulling rates, melt stability, and atmosphere conditions for each material system. Consequently, the present approach should be regarded as a general preparation concept rather than a universal solution applicable without further adjustments.

Nevertheless, the successful synthesis of high-quality single crystals across the investigated *A*_2_Zr_2_O_7_ compositions suggests that direct laser-heated Czochralski growth from mixed-oxide precursors may represent a valuable alternative to conventional precursor preparation for a wide range of refractory materials.

## 4 Conclusions

A preparation route for rare-earth zirconate single crystals based on laser-heated Czochralski growth directly from mixed-oxide precursors has been presented. The method eliminates the need for conventional synthesis of fully reacted polycrystalline feed rods while preserving the reproducibility and stability of the crystal-growth process.

Mechanically stable precursor rods were successfully prepared by homogenization, compaction, and low-temperature presintering of stoichiometric mixtures of rare-earth and zirconium oxides. Subsequent laser-heated pedestal growth yielded centimeter-sized single-crystalline ingots throughout the investigated *A*_2_Zr_2_O_7_ compositions, demonstrating that effective homogenization can be achieved directly within the molten zone.

Powder X-ray diffraction, Laue X-ray diffraction, electron microscopy, and energy-dispersive X-ray spectroscopy confirmed the formation of phase-pure, chemically homogeneous, and structurally well-defined single crystals. The refined structural parameters agree with previously reported data, indicating that direct crystal growth from mixed-oxide precursors does not alter the structural characteristics of the resulting materials.

The successful preparation of high-quality single crystals across the investigated *A*_2_Zr_2_O_7_ compositions demonstrates that direct laser-heated Czochralski growth from mixed-oxide precursors constitutes a practical alternative to conventional precursor processing. Beyond the *A*_2_Zr_2_O_7_ family, the methodology should be applicable to other refractory materials that can be grown from the melt and whose constituents remain stable under crystal-growth conditions.
